# Droughts and child health in Bangladesh

**DOI:** 10.1371/journal.pone.0265617

**Published:** 2022-03-21

**Authors:** Kien Le, My Nguyen

**Affiliations:** Faculty of Economics and Public Management, Ho Chi Minh City Open University, Ho Chi Minh City, Vietnam; Sam Houston State University, UNITED STATES

## Abstract

This paper investigates the extent to which in-utero exposure to droughts influences the health outcomes of Bangladeshi children in early childhood. Exploiting the plausibly exogenous deviations of rainfall from the location-specific norms, we find that deficient rainfall during the prenatal period is harmful to child health. Specifically, in-utero exposure to droughts decreases the height-for-age, weight-for-height, and weight-for-age z-scores by 0.10, 0.11, and 0.11 standard deviations among children under five years old, respectively. Our heterogeneity analyses reveal that the adverse health setbacks fall disproportionately on children of disadvantaged backgrounds. Exploring the differential effects by trimesters of exposure, we further show that experiencing droughts during the second and the third trimesters leaves injurious effects on early childhood health.

## 1. Introduction

Climate change has raised the frequency and intensity of extreme weather events such as deficient rainfall or droughts, posing substantial challenges to socio-economic development. At the macro level, prior studies show that deficient rainfall reduces national incomes and slows economic growth rates [1–3]. The lack of rainfall is also shown to induce conflict and political instability in various contexts [4, 5]. At the micro level, the profound impacts of deficient rainfall or drought are documented. In particular, droughts depress agricultural production, thus reducing household incomes from agriculture [6–8]. The prolonged dry spells induced by droughts raise the frequency of forest fire, whereas the disruption on water resources can interfere with the spawning habitats of fish and other aquatic animals, thus negatively affecting aquaculture [9]. Furthermore, droughts could leave adverse impacts on human development by reducing the investment in education and health [10, 11].

This paper investigates the extent to which in-utero exposure to droughts affects early childhood health in the context of Bangladesh. The paper makes three contributions to the literature. First, we evaluate the less tangible cost of an extreme weather event, drought in this case, to early human health, while other studies tend to focus on the more visible impacts, thus augmenting the current knowledge of the weather-economy relationship. Second, we examine the impacts of experiencing droughts in the intrauterine period in a different setting, i.e. Bangladesh. Therefore, the paper sheds additional light on the vestigial effects of early life shocks, providing meaningful implications for policy-making. This is relevant not only for Bangladesh but also for other agro-based countries where living standards are still low and the rural livelihoods are highly susceptible to adverse rainfall shocks such as droughts. Finally, our study also contributes to the debate on the relative importance of intrauterine exposure timing by showing evidence on the impacts of second and third trimester exposure to droughts.

To examine how a drought experience during the in-utero period influences the health outcomes of under five Bangladeshi children, we mainly rely on the Bangladesh Demographic and Health Survey supplemented with the Global Positing System components (DHS-GPS) and the Global Historical Climatology Network-monthly (GHCNm). The Bangladesh DHSGPS is a rich source of information on child anthropometric health measures as well as other important characteristics. The GHCNm offers rainfall data from land-based stations, which provides an accurate measurement of a particular location’s climatic indicators. As for the empirical strategy, our study exploits the plausibly exogenous deviations of rainfall from the location-specific norms to isolate the impacts of droughts that occur in the intrauterine period. The underlying assumption is that the occurrence of droughts is a quasi-random event across and within residential clusters, thus, in-utero exposure to droughts is also quasi-random.

Our study reaches the following findings. First, we find that in-utero exposure to droughts worsens the health outcomes of Bangladeshi under five children. Specifically, the height-forage, weight-for-height, and weight-for-age z-scores decline by 0.10, 0.11, and 0.11 standard deviations, respectively, if the child was exposed to droughts during the intrauterine period. These estimates respectively represent the reductions by 6.3, 11.0, and 6.3%, relative to the sample averages. Second, examining the heterogeneous impacts of droughts, our analyses reveal that the adverse health setbacks fall disproportionately on children born to low-educated mothers, those born to poor mothers, and those born to mothers living in rural areas. The findings underscore the susceptibility of children of disadvantaged socioeconomic backgrounds to adverse weather shocks such as droughts. Third, examining the differential effects by trimesters of exposure, we further show that experiencing droughts during the second and the third trimesters leaves injurious effects on early childhood health. Finally, our findings remain unchanged if alternate measures of child health and droughts are utilized.

Our study highlights the health costs of weather extremes such as droughts which tend to increase in both frequency and intensity as climate change evolves. Because the consequences of poor childhood health can persist to adulthood such as increased vulnerability to diseases, lower cognitive ability, and fewer earnings [12–15], a drought experience during the intrauterine period might be harmful to long-term human development. Therefore, our study calls for effective measures to mitigate the consequences of droughts. The government should subsidize programs that aim to provide financial and nutrition support for pregnant women in drought-prone areas. Health care intervention should be implemented to decrease pregnant women’s vulnerability to drought-related illnesses. Children of disadvantaged backgrounds such as those born to low educated mothers, poor mothers, and mothers living in rural areas should receive extra attention as they tend to be the most susceptible.

The paper proceeds as follows. Section 2 provides the overview of droughts in Bangladesh and summarizes related literature. Section 3 describes the data. Section 4 presents the empirical methodology. Section 5 discusses the results. Section 6 concludes the paper.

## 2. Overview of droughts in Bangladesh and literature review

### 2.1. Overview of droughts in Bangladesh

A high proportion of the Bangladeshi population is exposed to weather extremes with droughts being one of the most hazardous conditions [16, 17]. Rainfall deficiency is a leading cause of drought. In drought-affected regions, the annual average rainfall vary between 1,400 and 1,900 millimeters while the country-wide average is around 2,200 millimeters [17]. The authors further document that in drought-affected regions, annual average rainfall can sometimes fall below 1,000 millimeters, for example, to 793 millimeters in 2009. As climate change alters the variability of rainfall, the incidence of droughts in Bangladesh has become increasingly more intense and frequent, posing a substantial challenge to Bangladeshi society [18].

According to [19], the North and North-western areas in Bangladesh are particularly vulnerable. The Barind (upland of Northwestern part) is regarded as the most drought-prone area, which covers the most part of the greater Dinajpur, Rangpur, Pabna, Rajshahi, Chapai Nawabganj, Bogra, Joypurhat, and Naogaon districts [17]. Besides, the Southwestern part of Bangladesh also suffers from droughts but the severity is moderate. The drought-prone areas of the Southwestern part include Jhenaidah, Jessore, and Satkhira districts where droughts mainly occur during the dry season [17].

According to [16, 19], approximately 53% of the Bangladeshi population is affected by droughts. Droughts could destroy agricultural production, leading to food security, livelihood insecurity, and malnutrition [7, 8]. Droughts might also interfere with fish habitat and population, thus hindering fishing activities and disrupting aquacultural livelihood [9]. Droughts can further cause substantial economic loss for the livestock dependent population as droughts could create food scarcity for livestock and decrease their reproductive capacity [17]. Besides, droughts may lead to various health hazards such as dysentery and diarrhea [19].

### 2.2. Literature review

Our study is related to two strands of literature. The first strand of literature focuses on the important vestigial effects of in-utero shocks on child health. [20] present evidence for the devastating impacts of being exposed to a locust plague in utero on child health in Mali. The authors find that compared to non-exposed children, exposed children are substantially shorter for their age. Food price inflation is another shock that could harm child health as pointed out in [21, 22]. While [21] show that in-utero exposure raises the incidences of childhood stunting and underweight, [22] uncover the rising mortality risk among children under five years old in Ethiopia. Also relevant to this context, [23] reports the detrimental impacts of intrauterine exposure to food scarcity on child height. Besides, a number of studies regard armed conflict as an adverse shock that could threaten early childhood health, if it happens during the intrauterine period. Specifically, [24] shows that in-utero exposure to the protracted Afghan conflict adversely affects the height-for-age and weight-for-age of children. In the context of the Eritrea-Ethiopia conflict, [25] find that fetal exposure to war leads to lower height-for-age for children in both losing and winning sides. In a different setting, [26] uncover the substantial health setbacks among Ivorian children exposed to armed conflict. Our study contributes to this branch of literature by exploiting a weather-related event, deficient rainfall or drought, as an in-utero shock, to evaluate the persistent impacts of prebirth conditions.

The second line of study which our study also fits into explores the relationship between deficient rainfall conditions (or droughts) and child health. [27] detect faltering growth among rural Zimbabwean children in the aftermath of drought. Focusing on Kenya, [28] find that a drought experience worsens child health measured by mid-upper arm circumference. The authors also detect larger impacts on boys than girls. More recent studies also document a strong negative link between drought and child health. Particularly, the work of [11] shows that droughts are associated with worse child nutrition. [29] document that reduced rainfall has deleterious effects on child health in the form of increased incidences of illnesses such as diarrhea, cough, and fever. A few studies examine the impacts of droughts that occur in the in-utero period on child health and the findings are quite mixed. For instance, [30, 31] find that prenatal exposure to deficient rainfall does not have any effect on height-for-age and weight-for-age z-scores of Indonesian and Indian children, respectively. However, in the context of India, [32] detect negative impacts of in-utero exposure to drought on child’s weight-for-age. Our study adds to this literature by evaluating how deficient rainfall prior to birth can affect after-birth outcomes in a different context, i.e., Bangladesh. We also utilize all three commonly used anthropometric measures to capture child health, namely, height-for-age, weight-for-height, and weight-for-age, instead of looking at just one individual measure. Besides, we conduct an exhaustive heterogeneity analysis to identify the most vulnerable population group.

## 3. Data

### 3.1. Child health

We draw from the Bangladesh Demographic and Health Survey (DHS) for the data on health outcomes of under five Bangladeshi children. Child health is measured by anthropometric z-scores including height-for-age, weight-for-height, and weight-for-age. These health measures are calculated for children under five years old by their age and sex, based on the Centers for Disease Control and Prevention (CDC) Standard Deviation-derived Growth Reference Curves. The Growth Reference Curves are constructed based on the National Center for Health Statistics (NCHS)/CDC Reference Population. Height-for-age, weight-for-height, and weight-for-age z-scores are expressed as the number of standard deviations below or above the median of the international reference population. These anthropometric z-scores are commonly used measures that reflect the nutrition and growth status of children. Specifically, while height-for-age indicates long-run health status, weight-for-height indicates current health status [33]. In other words, a low height-for-age is induced by prolonged exposure to environmental stressors, whereas a low weight-for-height is brought about by insufficient nutrient intake and the disease infection in the short run. Weight-for-age is influenced by both height-for-age and weight-for-height [33].

For the purpose of our study, we employ the Bangladesh DHS waves with available information on anthropometric z-scores. We also keep children whose mothers always reside in the same place before conception. We further restrict our sample to DHS waves supplemented by the Global Positioning System components (DHS-GPS) in which participating Bangladeshi households are geo-referenced. In other words, DHS-GPS allows us to identify the latitude-longitude coordinates of the residential cluster (the lowest geographic level) the child’s household falls into. Given such detailed geographic information, we are able to extract the rainfall record of each residential cluster. With these restrictions, we end up with child data being taken from waves 3, 4, 5, and 6 of the Bangladesh DHS-GPS (corresponding to the periods 1993–1997, 1999–2004, 2007, and 2011).

### 3.2. Rainfall

**Fig 1:**
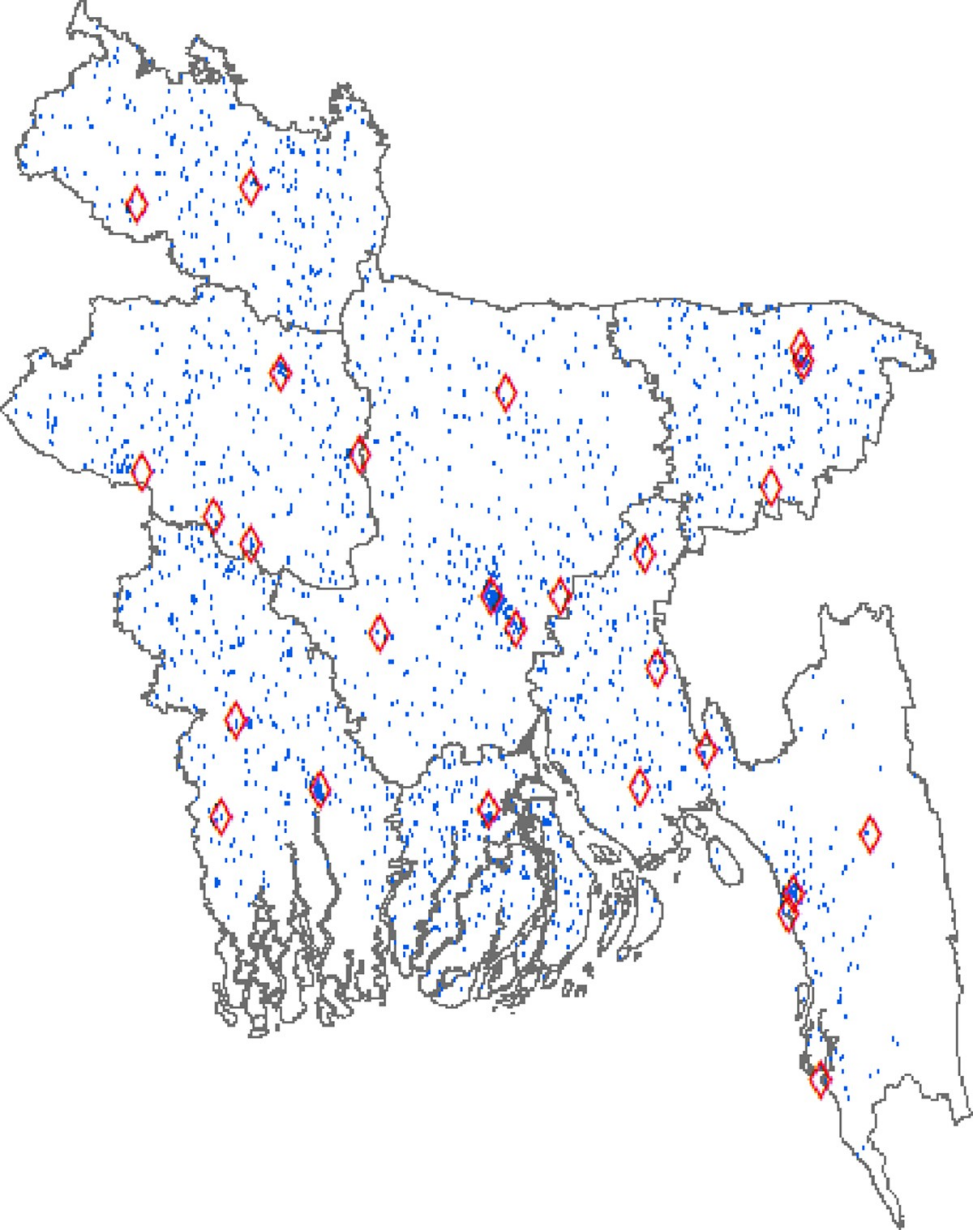
Geographic distribution of rainfall stations across Bangladesh. Rainfall stations are illustrated by red diamonds. Residential clusters are in blue dots. The figure is created using ArcMap. The data used to construct the map were obtained from the following open access sources: Data for rainfall stations are from the Global Historical Climatology Network monthly and downloadable from https://ncei.noaa.gov/products/land-based-station/global-historical-climatology-network-monthly. Data for residential clusters are from the Bangladesh Demographic and Health Survey and downloadable from https://dhsprogram.com/data/available-datasets.cfm. The base map layer is from the Database of Global Administrative Areas and downloadable from https://gadm.org/download_country.html.

Our rainfall data for Bangladesh are retrieved from the Global Historical Climatology Network monthly (GHCNm). The GHCNm is distributed by the National Centers for Environmental Information. The data provide a rich record of rainfall collected monthly from stations across Bangladesh during the 1990–2015 period. It is also worth noting that this period is chosen because it covers the entire reference period of Bangladesh DHS-GPS. Next, we assign rainfall data for each residential cluster based on the records from the closest station [34, 35]. We also adopt a different method of assigning rainfall data where we take the average of rainfall from closeby stations (Section 5.4: Panels C and D of Table 7) to test for the sensitivity of our results. Fig 1 illustrates the distribution of rainfall stations (red diamonds) and residential clusters (blue dots) across Bangladesh.

One disadvantage of the GHCNm is that several stations failed to deliver rainfall records in some months. If months with missing data are correlated with extreme rainfall events, our estimates can be biased (e.g. hypothetically, if droughts were more likely to occur in January and missing data concentrated in January simultaneously, our estimates could be biased). To assess such a possibility, in Fig A1 in S1 Appendix, we plot the distribution of months with missing rainfall data in the GHCNm. Months with missing rainfall data seem to be evenly distributed, suggesting that such missing values are quite random and not correlated with rainfall. Thus, we supplement the rainfall information from the GHCNm with data from the Climatic Research Unit Time Series where rainfall level is estimated for each 0.5°×0.5° area by using the angular distance-weight average from the surrounding stations, addressing the issue of missing record from one particular station.

Since we know the child’s month and year of birth, we can further determine the rainfall level the child was exposed to in his/her residential cluster during the in-utero period. In measuring rainfall, we follow the literature by looking at the deviation of rainfall from the long-run local average [30, 35, 36]. Specifically, we calculate the deviation of rainfall within the nine-month intrauterine period from the long-run mean of total rainfall during that period for the child’s residential cluster. To do so, we first subtract the cluster’s long-run average rainfall from total rainfall in the child’s cluster during the nine months of gestation, then divide by the cluster’s long-run standard deviation of rainfall. The long-run local average and standard deviation are for the period between 1990 and 2015. Mathematically put, the in-utero rainfall deviation (*IURD*) is given by,

$$IURD=\frac{TR-LRAR}{LRSDR}$$

where *TR* denotes total rainfall in the child’s cluster within the nine-month intrauterine period. The terms *LRAR* and *LRSDR* stand for long-run local average and standard deviation of rainfall in the child’s cluster between 1990 and 2015. Because we are interested in examining how in-utero exposure to droughts affects early childhood health, we proceed to construct a measure of drought as rainfall falling below the location-specific norm by at least one standard deviation [37]. In other words, our main explanatory variable, *Drought*, takes the value of one if *IURD ≤ −1*, and zero otherwise.

### 3.3. Estimation sample

Our estimation sample consists of approximately 28,600 children. Descriptive statistics for outcome and independent variables are presented in Panels A and B, respectively. According to Panel A, height-for-age, weight-for-height, and weight-for-age z-scores, on average, are -1.6, -1.0, and -1.7 standard deviations, respectively. The negative values reflect that the average health measures of Bangladeshi children are below the world median values which include children from higher income countries. Moving to Panel B, approximately 10.2% of Bangladeshi children in our sample were exposed to droughts during the in-utero period. Mothers complete roughly 4.8 years of education on average. The mean value of mother’s age at birth is 23.96 years. Around 71% of households live in rural areas and 92% of households are headed by a male. The average value of the wealth index is 2.9. Approximately 51% of children are male. The mean values of child’s age (in months) and birth order are 29.4 and 2.6, respectively. The fraction of singleton birth is 98.7%.

As a preliminary check, we plot the differences in height-for-age, weight-for-height, and weight-for-age z-scores between children prenatally exposed to droughts and those unexposed, by year of birth. In Fig A2 in S1 Appendix, for each birth year, we calculate the average difference in height-for-age z-scores between exposed and unexposed children, which are represented by hollow circles. The weight-for-height and weight-for-age z-scores are illustrated in Figs A3 and A4 in S1 Appendix, respectively. The red dashed line represents the mean value of the anthropometric difference. Because children prenatally exposed to droughts tend to have lower anthropometric z-scores than those prenatally unexposed, on average, the difference value is negative.

## 4. Empirical methodology

To investigate the effects of in-utero exposure to droughts on child health, we estimate the following regression model,

$$Y_{irtm}=\beta_{0}+\beta_{1}{Drought}_{rtm}{+\lambda }_{r}{+\gamma }_{t}{+\delta_{m}+X\prime }_{irtm}\Omega {+\epsilon }_{irtm}$$

where the subscripts *i*, *r*, *t*, and *m* stand for child, residential cluster, birth year, and birth month respectively. A residential cluster is a geo-referenced place of residence where the child resides. The variable *Y_irtm_* represents the outcome of interest, including three anthropometric (height-for-age, weight-for-height, weight-for-age) z-scores. Our main explanatory variable is *Drought_rtm_* that receives the value of one if total rainfall in the child’s cluster during the nine months of gestation are at least one standard deviation below the cluster’s long-run local mean of rainfall, and zero otherwise.

We also denote by *λ_r_*, *γ_t_*, and *δ_m_* residential cluster, birth year, and birth month fixed effects, respectively. The vector *X*′_*irtm*_ includes mother and child characteristics, including: (i) mother’s years of education, mother’s age at birth, mother’s age at birth squared, household head gender, household wealth index, whether the household resides in a rural area, and (ii) child’s gender, child’s birth order, child’s age in months, child’s age in months squared, as well as whether the child is a singleton birth. Finally, *ϵ_irtm_* stands for the error term. Standard errors throughout the paper are clustered at the residential cluster level.

Our coefficient of interest is *β*_1_ that captures the effects of fetal exposure to droughts on early childhood health. The assumption behind this empirical framework is that the occurrence of droughts is a quasi-random event across and within residential clusters, thus, in-utero exposure to droughts is also quasi-random. The assumption is likely to hold as prior studies show that the deviations of rainfall from the long-term local average are exogenous [30, 36, 38]. Therefore, the estimate of *β*_1_ is unbiased and reflects the effects of droughts during the intrauterine period on child health.

## 5. Results

### 5.1. Main results

The estimated impacts of intrauterine exposure to droughts on child health are presented in Table 1. Each column is a separate regression and the column heading indicates the dependent variable. All regressions control for mother’s characteristics (mother’s years of education, mother’s age at birth, mother’s age at birth squared, household head gender, household wealth index, whether the household resides in a rural area), child’s characteristics (child’s gender, child’s birth order, child’s age in months, child’s age in months squared, as well as whether the child is a singleton birth), birth month, birth year, and residential cluster fixed effects.

**Table 1:** Prenatal exposure to droughts and child health—Main results.

	Height-for-Age	Weight-for-Height	Weight-for-Age
	Z-Score	Z-Score	Z-Score
	(1)	(2)	(3)
Drought	-0.096*** (0.034)	-0.112*** (0.027)	-0.109*** (0.028)
Observations	28,571	28,571	28,571
Mother Characteristics	X	X	X
Child Characteristics	X	X	X
All Fixed Effects	X	X	X

Note

*p<0.1

**p<0.05

***p<0.01. Each column represents the coefficients in a separate regression. The column headings indicate dependent variables. Mother Characteristics consist of mother’s age at birth, mother’s age at birth squared, mother’s years of education, household wealth index, household head gender, whether the household resides in a rural area. Child Characteristics consist of child’s age in months, child’s age in months squared, child’s gender, child’s birth order, as well as whether the child is a singleton birth. All Fixed Effects consist of residential cluster, birth month, and birth year fixed effects. Robust standard errors are clustered at the residential cluster level.

Evident from Column 1, exposure to droughts during the in-utero period reduces child’s height-for-age z-score by approximately 0.10 standard deviations. Experiencing droughts throughout the nine months in gestation also makes Bangladeshi children 0.11 standard deviation thinner for their height and 0.11 standard deviation thinner for their age (Columns 2 and 3). Taking the mean values of the corresponding anthropometric measures in Table 2 as the benchmarks, these estimates represent the reductions by 6.3, 11.0, and 6.3% in height-for-age, weight-for-height, and weight-for-age z-scores.

**Table 2:** Summary statistics.

	Mean	SD	Obs.
	(1)	(2)	(3)
**Panel A: Outcome Variables**			
Height-for-Age Z-Score	-1.577	1.340	28,591
Weight-for-Height Z-Score	-0.996	1.016	28,591
Weight-for-Age Z-Score	-1.742	1.114	28,591
**Panel B: Independent Variables**			
Drought	0.102	0.303	28,591
Mother’s Education	4.836	4.704	28,582
Mother’s Age at Birth	23.96	5.889	28,591
Rural Area	0.709	0.454	28,591
Male Household Head	0.922	0.268	28,591
Wealth Index	2.942	1.434	23,987
Male Child	0.508	0.500	28,591
Child’s Age in Month	29.42	17.17	28,591
Child’s Birth Order	2.627	1.756	28,591
Singleton Birth	0.987	0.113	28,591

### 5.2. Heterogeneity

We proceed to explore the heterogeneity in the impacts of droughts on child health along the lines of mother’s education, mother’s wealth, and mother’s place of residence. The estimating results are provided in Table 3. In each panel, each column is a separate regression and the column heading indicates the dependent variable. The panel name presents the dimensions of heterogeneity.

**Table 3:** Prenatal exposure to droughts and child health—Heterogeneity analysis.

	Height-for-Age	Weight-for-Height	Weight-for-Age
	Z-Score	Z-Score	Z-Score
	(1)	(2)	(3)
**Panel A: Low-education Mothers**			
Drought	-0.199*** (0.052)	-0.110*** (0.040)	-0.179*** (0.040)
Observations	12,950	12,950	12,950
**Panel B: High-education Mothers**			
Drought	-0.011 (0.045)	-0.098** (0.042)	-0.040 (0.041)
Observations	15,285	15,285	15,285
**Panel C: Poor Mothers**			
Drought	-0.086 (0.054)	-0.118*** (0.043)	-0.107*** (0.039)
Observations	9,865	9,865	9,865
**Panel D: Non-Poor Mothers**			
Drought	-0.039 (0.054)	-0.078* (0.045)	-0.033 (0.048)
Observations	13,853	13,853	13,853
**Panel E: Rural Mothers**			
Drought	-0.103*** (0.038)	-0.129*** (0.030)	-0.127*** (0.031)
Observations	20,266	20,266	20,266
**Panel F: Urban Mothers**			
Drought	-0.065 (0.070)	-0.031 (0.059)	-0.030 (0.060)
Observations	8,305	8,305	8,305
Mother Characteristics	X	X	X
Child Characteristics	X	X	X
All Fixed Effects	X	X	X

Note

*p<0.1

**p<0.05

***p<0.01. Each column represents the coefficients in a separate regression. The column headings indicate dependent variables. Mother Characteristics consist of mother’s age at birth, mother’s age at birth squared, mother’s years of education, household wealth index, household head gender, whether the household resides in a rural area. Child Characteristics consist of child’s age in months, child’s age in months squared, child’s gender, child’s birth order, as well as whether the child is a singleton birth. All Fixed Effects consist of residential cluster, birth month, and birth year fixed effects. Robust standard errors are clustered at the residential cluster level.

First, we examine if droughts differentially affect children born to low-educated mothers and highly educated mothers. Low-educated mothers are those who did not complete primary education. Likewise, highly educated mothers are those with at least primary education. We find that in-utero exposure to droughts decreases the height-for-age, weight-for-height, and weight-for-age z-scores of children born to low-educated mothers by approximately 0.20, 0.11, and 0.18 standard deviations, respectively (Panel A). All estimates are statistically distinct from zero. For children born to highly educated mothers, two out of three estimates lose statistical significance, and the magnitude of the estimates declines by roughly 94, 11, 78% for height-for-age, weight-for-height, and weight-for-age z-scores, respectively, compared to those of children born to low-educated mothers (Panel B). The results suggest that the adverse impacts of drought during the intrauterine period fall disproportionately on children of low-educated mothers. To put it differently, maternal education could serve as a buffer against the health setbacks induced by weather shocks such as droughts [39].

Second, we proceed to examine the heterogeneous impacts of in-utero exposure to droughts by mother’s wealth in Panels C and D. Poor mothers are defined as those coming from households which have wealth index falling to the bottom and the next bottom quintiles of the within-country distribution. Non-poor mothers are those in households which have wealth index in the middle, upper middle, and top quintiles of the within-country distribution. For children of poor mothers, experiencing droughts in the intrauterine period leads to the reductions in height-for-age, weight-for-height, and weight-for-age z-scores by 0.09, 0.12, and 0.11 standard deviations, respectively (Panel C). For children of non-poor mothers, in-utero exposure to droughts exerts negative impacts on all three measures of child health but the magnitude of the impacts fall by approximately 56, 33, and 73%, respectively, compared to the impacts on children of poor mothers. The results highlight the importance of a more advantaged socio-economic background in conditioning the detrimental repercussions of negative shocks such as droughts.

Finally, in Panels E and F, we explore the heterogeneity in the impacts of droughts along the lines of mother’s place of residence. We find that fetal exposure to droughts reduces height-for-age, weight-for-height, and weight-for-age z-scores for children born to rural mothers by 0.1, 0.13, and 0.13 standard deviations, respectively (Panel E). As for children born to urban mothers, experiencing droughts during the intrauterine period makes children 0.07, 0.03, and 0.03 standard deviations shorter for their age, thinner for their height, and thinner for their age, respectively (Panel F). The estimates for children of mothers living in urban areas are statistically indistinguishable from zero and 30–77% smaller than the corresponding estimates for children born to mothers living in rural areas. The results suggest that rural children are especially vulnerable to adverse shocks such as droughts.

In the S1 Appendix, we further examine if the impacts of in-utero exposure to droughts differ by child’s gender and birth order. Evident from Panels A and B of Table A1 in S1 Appendix, both male and female children are adversely affected by droughts and the magnitudes of the impact do not seem to differ. As shown in Panels C and D of Table A1 in S1 Appendix, there is not enough evidence for the heterogeneous effects of droughts by birth order as the estimates are close in magnitude for first-birth children and later birth ones.

### 5.3. Trimester analysis

We detected negative impacts of prenatal exposure to droughts on child’s anthropometric z-scores. In this section, we further explore if exposure at different trimesters of pregnancy affects child health differently. To do so, we replace the single indicator for in-utero drought exposure with three indicators for drought exposure during each of the three trimesters (1st Trimester Drought, 2nd Trimester Drought, 3rd Trimester Drought). The estimating results are reported in Table 4.

**Table 4:** Prenatal exposure to droughts and child health—Trimester analysis.

	Height-for-Age	Weight-for-Height	Weight-for-Age
	Z-Score	Z-Score	Z-Score
	(1)	(2)	(3)
1st Trimester Drought	0.028 (0.030)	-0.007 (0.023)	0.010 (0.024)
2nd Trimester Drought	-0.060** (0.029)	-0.086*** (0.025)	-0.091*** (0.023)
3rd Trimester Drought	-0.015 (0.030)	-0.120*** (0.025)	-0.090*** (0.024)
Observations	28,571	28,571	28,571
Mother Characteristics	X	X	X
Child Characteristics	X	X	X
All Fixed Effects	X	X	X

Note

*p<0.1

**p<0.05

***p<0.01. Each column represents the coefficients in a separate regression. The column headings indicate dependent variables. Mother Characteristics consist of mother’s age at birth, mother’s age at birth squared, mother’s years of education, household wealth index, household head gender, whether the household resides in a rural area. Child Characteristics consist of child’s age in months, child’s age in months squared, child’s gender, child’s birth order, as well as whether the child is a singleton birth. All Fixed Effects consist of residential cluster, birth month, and birth year fixed effects. Robust standard errors are clustered at the residential cluster level.

We find that exposure to droughts in the second trimester adversely affects all three anthropometric z-scores as the estimates are both statistically and economically significant. Drought exposure in the third trimester is harmful to child’s weight-for-height and weight-for-age, but not height-for-age z-scores. There is not enough evidence for the impacts of first trimester exposure. Taken together, experiencing droughts during the second and the third trimesters leaves injurious effects on early childhood health. The results are consistent with prior studies which also emphasize the health threat induced by the last two trimester exposure to negative shocks [40–43].

### 5.4. Other measures

In this section, we attempt to test the robustness of our main results by using different measures of outcome and explanatory variables. The estimating results are displayed in Tables 5–7. In these tables, each column is a separate regression and the column heading indicates the dependent variables.

**Table 5:** Prenatal exposure to droughts and child health—Other outcomes 1.

	Stunting	Wasting	Underweight
	(1)	(2)	(3)
Drought	0.047*** (0.013)	0.028*** (0.010)	0.053*** (0.013)
Observations	28,571	28,571	28,571
Mother Characteristics	X	X	X
Child Characteristics	X	X	X
All Fixed Effects	X	X	X

Note

*p<0.1

**p<0.05

***p<0.01. Each column represents the coefficients in a separate regression. The column headings indicate dependent variables. Mother Characteristics consist of mother’s age at birth, mother’s age at birth squared, mother’s years of education, household wealth index, household head gender, whether the household resides in a rural area. Child Characteristics consist of child’s age in months, child’s age in months squared, child’s gender, child’s birth order, as well as whether the child is a singleton birth. All Fixed Effects consist of residential cluster, birth month, and birth year fixed effects. Robust standard errors are clustered at the residential cluster level.

**Table 6:** Prenatal exposure to droughts and child health—Other outcomes 2.

	Height-for-Age	Weight-for-Height	Weight-for-Age
	Percentile	Percentile	Percentile
	(1)	(2)	(3)
Drought	-0.723 (0.613)	-2.090*** (0.638)	-1.298*** (0.506)
Observations	28,571	28,571	28,571
Mother Characteristics	X	X	X
Child Characteristics	X	X	X
All Fixed Effects	X	X	X

Note

*p<0.1

**p<0.05

***p<0.01. Each column represents the coefficients in a separate regression. The column headings indicate dependent variables. Mother Characteristics consist of mother’s age at birth, mother’s age at birth squared, mother’s years of education, household wealth index, household head gender, whether the household resides in a rural area. Child Characteristics consist of child’s age in months, child’s age in months squared, child’s gender, child’s birth order, as well as whether the child is a singleton birth. All Fixed Effects consist of residential cluster, birth month, and birth year fixed effects. Robust standard errors are clustered at the residential cluster level.

**Table 7:** Prenatal exposure to droughts and child health—Other explanatory.

	Height-for-Age	Weight-for-Height	Weight-for-Age
	Z-Score	Z-Score	Z-Score
	(1)	(2)	(3)
**Panel A: Standardized Measure**			
Standardized Rainfall Deficiency	-0.094*** (0.023)	-0.057*** (0.018)	-0.079*** (0.019)
Observations	28,571	28,571	28,571
**Panel B: Percentage Measure**			
Percentage Lack of Rainfall	-0.433*** (0.138)	-0.303*** (0.104)	-0.407*** (0.109)
Observations	28,571	28,571	28,571
**Panel C: Drought at the District Level**			
District Drought	-0.080*** (0.026)	-0.086*** (0.020)	-0.090*** (0.020)
Observations	28,571	28,571	28,571
**Panel D: 2-Station Average Measure**			
Drought	-0.100*** (0.034)	-0.112*** (0.029)	-0.114*** (0.029)
Observations	28,571	28,571	28,571
**Panel E: Drought and Flood**			
Drought	-0.095*** (0.034)	-0.108*** (0.027)	-0.105*** (0.028)
Flood	-0.106** (0.045)	-0.178*** (0.038)	-0.314*** (0.038)
Observations	28,571	28,571	28,571
Mother Characteristics	X	X	X
Child Characteristics	X	X	X
All Fixed Effects	X	X	X

Note

*p<0.1

**p<0.05

***p<0.01. Each column represents the coefficients in a separate regression. The column headings indicate dependent variables. Mother Characteristics consist of mother’s age at birth, mother’s age at birth squared, mother’s years of education, household wealth index, household head gender, whether the household resides in a rural area. Child Characteristics consist of child’s age in months, child’s age in months squared, child’s gender, child’s birth order, as well as whether the child is a singleton birth. All Fixed Effects consist of residential cluster, birth month, and birth year fixed effects. Robust standard errors are clustered at the residential cluster level.

First, we replace three anthropometric z-scores with the three nutrition indicators as the dependent variables. Following [33], we define Stunting, Wasting, and Underweight as indicators for height-for-age, weight-for-height, and weight-for-age z-scores being less than -2, respectively. Evident from Table 5, in-utero exposure to droughts elevates the probabilities of being stunted, wasted, and underweight by 4.7, 2.8, and 5.3 percentage points, respectively. Overall, experiencing droughts during the intrauterine period is harmful to child health. In other words, our conclusion remains unchanged if alternate measures of child health are utilized.

Second, instead of using the z-score classification system to express our outcome anthropometric measures, we rely on the percentile system. Particularly, height-for-age, weight-for-height, and weight-for-age percentiles reflect the ranking of the child’s anthropometric measures among the reference population. According to Table 6, in-utero exposure to droughts reduces height-for-age, weight-for-height, and weight-for-age rankings by 0.72, 2.09, and 1.30 percentiles, respectively. In other words, using the percentile system further corroborates our main findings with the z-score system.

Besides, we employ child height in centimeters and child weight in kilograms as our outcome variables and re-estimate the models. Evident from Table A2 in S1 Appendix, being prenatally exposed to droughts decreases child height and weight by 0.43 centimeters and 0.18 kilograms, respectively. Estimates are statistically distinct from zero. Here, we carefully note that the problem with raw centimeters and kilograms is that they are not unified across age and gender (e.g. 1 kg difference is not very severe for children aged five but it is a huge value for newborns). The anthropometric z-scores are preferred in the literature because they further account for age and gender.

Finally, we employ different measures of droughts to investigate the impacts on child health. The estimating results are reported in Table 7. Specifically, in Panel A, we replace the single drought indicator with a standardized measure of deficient rainfall (Standardized Rainfall Deficiency). Recall that to calculate the in-utero rainfall deviation (*IURD*), we first take the differences between total rainfall in the child’s cluster during the nine months of gestation and the cluster’s long-run average, then divided by the cluster’s long-run standard deviation (as in the equation in Section 3). Our measure of Standardized Rainfall Deficiency equals the absolute value of in-utero rainfall deviation (*IURD*) if the rainfall deviation is negative, and zero otherwise. To put it differently, Standardized Rainfall Deficiency reflects the rainfall falling below the location-specific norm, i.e., negative rainfall anomalies. Evident from Panel A, as negative rainfall anomalies in the prenatal period increases by one standard deviation, the child’s height-for-age, weight-for-height, and weight-for-age z-scores decrease by 0.09, 0.06, and 0.08 standard deviations, respectively.

In Panel B, we employ the percentage measure of deficient rainfall. Particularly, we calculate the percentage in-utero rainfall deviation (PIURD) by taking the differences between total rainfall in the child’s cluster during the nine months of gestation and the cluster’s long-run average, then divided by the cluster’s long-run average. Our Percentage Lack of Rainfall measure equals the absolute value of the percentage in-utero rainfall deviation if PIURD is negative, and zero otherwise. By construction, the Percentage Lack of Rainfall also captures deficient rainfall (i.e. negative rainfall anomalies). As shown in Panel B, a 10 percentage point increase in negative rainfall anomalies during the intrauterine period reduces Bangladeshi children’s height-for-age, weight-for-height, and weight-for-age z-scores by 0.04, 0.03, and 0.04 standard deviations, respectively. In brief, employing alternative measures of droughts leaves our conclusions unchanged.

Recall that in the main analysis, we define drought at the residential cluster level. In other words, we calculate the deviation of rainfall relative to the long-run local average the child was exposed to in his/her residential cluster during the in-utero period. Because the GPS coordinates associated with the residential clusters in the DHS-GPS data are randomly displaced to reduce disclosure risk [44], making our drought measure suffer from random measurement error. As a result, our estimates could be biased towards zero.

In this section, we test the robustness of our results by defining drought at the district level which is more aggregate than the residential cluster level. In particular, if there is one station in the district, we use rainfall data from that station. If there are more than two stations in the district, the average value of rainfall from those stations is assigned to the district. If there is no station in the district, we take the rainfall data from the nearest station to the district. Rainfall deviation and drought are calculated in a manner analogous to Section 3.2 except that it is conducted at the district instead of the residential cluster level. As shown in Panel C of Table 7, we still find adverse impacts of in-utero exposure to droughts on all three anthropometric z-scores. Estimates are close in magnitude to the main results in Table 1. To put it differently, defining drought at the district level does not largely affect our results.

Next, we adopt another method of measuring rainfall. Instead of assigning rainfall data in the GHCNm for each residential cluster based on the records from the closest station, we take the average of rainfall from the two nearest stations to the cluster. Drought is still defined at the residential cluster level in an analogous manner as in Section 3.2. As shown in Panel D of Table 7, we still find that in-utero exposure to droughts has adverse impacts on child health. Estimates are close in magnitude and significant level compared to our main results.

Finally, it is reported that excessive rainfall or flooding is regarded as a serious problem for Bangladesh. For example, Bangladesh faced one of the most extensive floods on record in July 1998, which was followed by another major flood in Jun 2004. Therefore, in the final robustness test, we account for the exposure to floods in our regression. Specifically, the variable Flood is an indicator taking the value of one if the child was in utero during the July 1998 and June 2004 floods, and zero otherwise. As shown in Panel E of Table 7, in-utero exposure to droughts and floods worsens child health measured by the three anthropometric z-scores. Estimates are negative and statistically significant at conventional levels, suggesting that the impacts of droughts are not driven by floods.

### 5.5. Discussion

Collectively, we have presented compelling evidence on the impacts of in-utero exposure to droughts on the health outcomes of Bangladeshi children. Specifically, experiencing droughts during the intrauterine period makes children 0.10, 0.11, and 0.11 standard deviations shorter for their age, thinner for their height, and thinner for their age, respectively. Taking the mean values of the anthropometric measures as the benchmarks, these estimates represent the reductions by 6.3, 11.0, and 6.3% in height-for-age, weight-for-height, and weight-for-age z-scores, respectively. Exploring the heterogeneous impacts of droughts, our analyses reveal that the adverse health setbacks fall disproportionately on children born to low-educated mothers, those born to poor mothers, and those born to mothers living in rural areas. The findings highlight the vulnerability of children of disadvantaged socioeconomic backgrounds to droughts. Examining the differential effects by trimesters of exposure, we further show that experiencing droughts during the second and the third trimesters leaves injurious effects on early childhood health.

Our findings are consistent with prior studies on the negative relationship between droughts and child health. Specifically, it is documented that droughts raise illness incidences and worsen various dimensions of child nutrition [11, 28, 29]. Our study also sheds additional evidence on how droughts occurring in the in-utero period can be linked to early childhood health. [30] do not detect the impact of prenatal exposure to deficient rainfall on the height-for-age of Indonesian children. [31] find that droughts occurring in the prenatal period do not affect the weight-for-age of Indian children. Also in the context of India, [32] show that in-utero exposure to drought leads to an approximately 0.1 standard deviation decrease in child’s weight-for-age, which is close to our estimate.

Besides, our study also complements the literature on the persistent impacts of in-utero shocks. Specifically, in-utero exposure to adverse shocks such as locust plague, food price inflation, food scarcity worsens child nutrition [20, 21, 23]. Experiencing armed conflicts during the prenatal period is also reported to leave deleterious impacts on early childhood health [24–26, 45]. Our study adds to this literature by exploiting drought as an adverse in-utero shock to evaluate the persistent effects of prenatal conditions. Furthermore, our findings are in accordance with studies emphasizing the importance of second and third trimester exposure to child outcomes [40–42].

There could be multiple channels through which in-utero exposure to droughts could adversely affect child health. First, it is possible that rainfall deficient conditions are associated with elevated incidences of vector-borne diseases and various drought-related illnesses [46–48]. The increased prevalence of diseases could harm the mother’s health, thus, impairing fetal growth and development [49, 50]. To the extent that poor infant health might raise the risk of poor health in early childhood, in-utero exposure to droughts can worsen early childhood health through the infliction of diseases during the intrauterine period. Second, another potential pathway to the impacts of droughts is the reduction in income and nutrition. It is documented that deficient rainfall depresses incomes from various sources such as agriculture, forestry, as well as aquaculture [7–9]. Lower incomes make it difficult for mothers to acquire adequate nutrition during pregnancy, thus raising the risks of fetal growth retardation and low birth weight [51, 52]. Since poor infant health might leave critical consequences to child health in later years, a drought experience during the in-utero period could be harmful to health and development outcomes of children. Third, droughts might raise the labor demand for women. As pointed out in [53, 54], pregnant women may have to travel a long distance to fetch water for agricultural activities and to collect water for drinking as well as cooking. Engagement in physically demanding tasks can considerably strain pregnant women, thus decreasing birth weight and child health [55]. In other words, experiencing droughts during the prenatal period could adversely affect child health through altering pregnant women’s time use.

As climate change increases the frequency and intensity of weather extremes such as droughts [56], our study underscores the health costs of climate change. To the extent that poor health in early childhood might lead to poorer physical and cognitive development, as well as declining productivity over the life cycle [12–15], being prenatally exposed to droughts could be devastating to long-term human development. As a result, our study calls for effective measures to mitigate the consequences of droughts. Subsidizing programs that aim to provide financial and nutrition support to pregnant women in drought-prone areas is justified. The longer-run financial solution should involve the development of adaptive strategies so that household livelihood becomes less dependent on weather events. Besides, health interventions intended to protect pregnant women from drought-related diseases are important to minimize the negative health impacts on the fetuses. Extra attention should be given to children from disadvantaged backgrounds such as those born to low-educated mothers, poor mothers, and mothers living in rural areas, as they tend to be the most vulnerable.

## 6. Conclusion

This paper investigates the extent to which in-utero exposure to droughts influences early childhood health in the context of Bangladesh. The data used in this study come from the Bangladesh Demographic and Health Survey supplemented with the Global Positing System components (DHS-GPS), the Global Historical Climatology Network-monthly (GHCNm), and the Climatic Research Unit Time Series (CRUTS) [57–60]. The Bangladesh DHS-GPS supplies details on child anthropometric measures as well as other characteristics. We mainly draw from the station-based GHCNm for the rainfall (drought) data. Because some GHCNm stations have missing rainfall data for some months, we supplement the GHCNm with the CRUTS. To identify the impacts of interest, we exploit the plausibly exogenous deviations of rainfall from the location-specific norms. Our empirical framework rests on the assumption that the occurrence of droughts is a quasi-random event across and within residential clusters, thus, in-utero exposure to droughts is also quasi-random.

Our study reaches the following findings. First, we find that fetal exposure to droughts leads to poorer health outcomes in early childhood. Specifically, Bangladeshi under five children are 0.10, 0.11, and 0.11 standard deviations shorter for their age, thinner for their height, and thinner for their age, respectively, if they experienced droughts during the intrauterine period. These estimates respectively represent the reductions by 6.3, 11.0, and 6.3% in height-for-age, weight-for-height, and weight-for-age z-scores, relative to the sample averages. Second, we detect heterogeneous impacts of a drought experience during gestation for various subgroups. Particularly, children of low-educated mothers, poor mothers, and mothers living in rural areas are severely hit by droughts that occur in the in-utero period while children of highly educated mothers, non-poor mothers, and mothers living in urban areas are hardly affected. Third, exploring the potentially differential impacts by exposure timing, our analyses reveal that exposure during the second and the third trimesters leaves injurious effects on early childhood health while there is not enough evidence for the effects of first trimester exposure. Finally, our findings on the impacts of in-utero exposure to droughts on early childhood health are robust to alternate measures of child health as well as various definitions of droughts.

Given the long-lasting impacts of poor health in early life on physical health, cognitive development, and productivity [12–15], the findings of our study imply that fetal exposure to droughts could be deleterious to human development in the long run. Effective measures should be implemented to mitigate the consequences of weather extremes such as droughts which could potentially increase in both frequency and intensity in light of climate change [56]. Financial and nutrition support for pregnant women in drought-prone areas might alleviate the adverse impacts on the health outcomes of their offspring. Government should also consider subsidizing health care for pregnant women due to the increased risks of diseases induced by droughts. Priorities should be given to children of disadvantaged backgrounds (those born to low-educated mothers, poor mothers, and mothers living in rural areas) since they tend to be the most vulnerable.

## Supporting information

S1 Appendix (DOCX)
