# Supplementary material for: Droughts and child health in Bangladesh
Source: PLoS One. 2022 Mar 21;17(3):e0265617. doi: 10.1371/journal.pone.0265617 (PMC8936449; doi:10.1371/journal.pone.0265617)
Supplement: S1 Appendix — (DOCX) [file pone.0265617.s001.docx]

**S1 Appendix.**

Figure A1: Distribution of Months with Missing Rainfall Information in the GHCNm

**
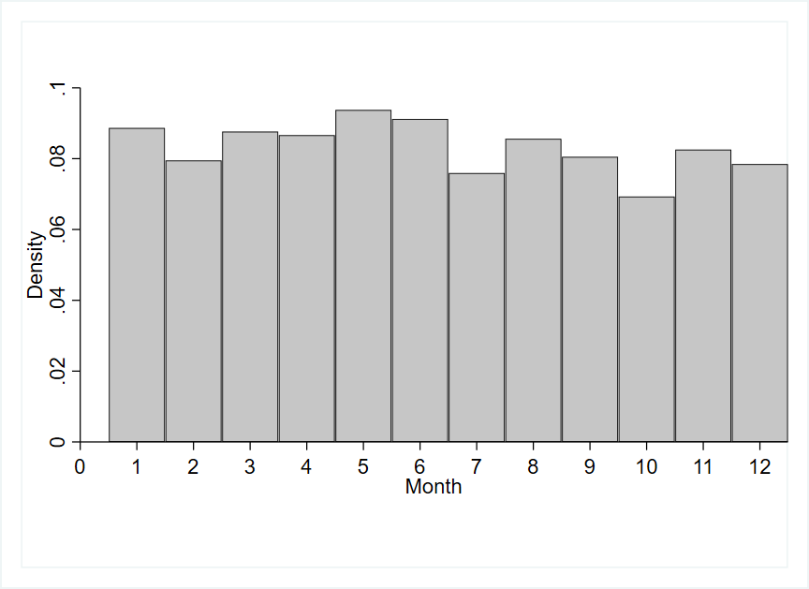
**

Note: This figure plots the distribution of months with missing rainfall data in the GHCNm

**Figure A2: Height-for-age Differences by Birth Year**

**
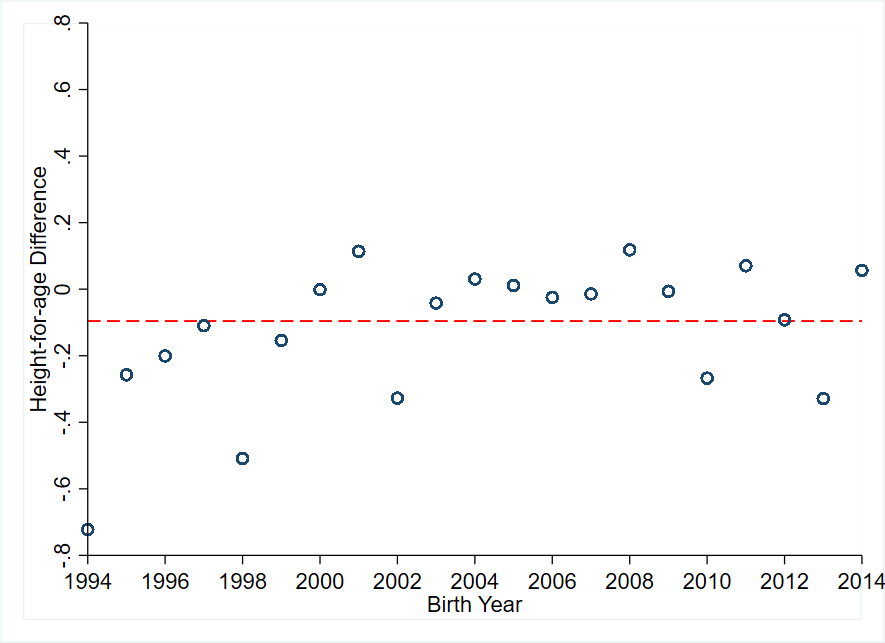
**

Note: For each birth year, the hollow circle represents the average difference in height-for-age z-scores between children prenatally exposed to droughts and those unexposed. The red dashed line represents the mean value of the height-for-age difference.

**Figure A3: Weight-for-height Differences by Birth Year**

**
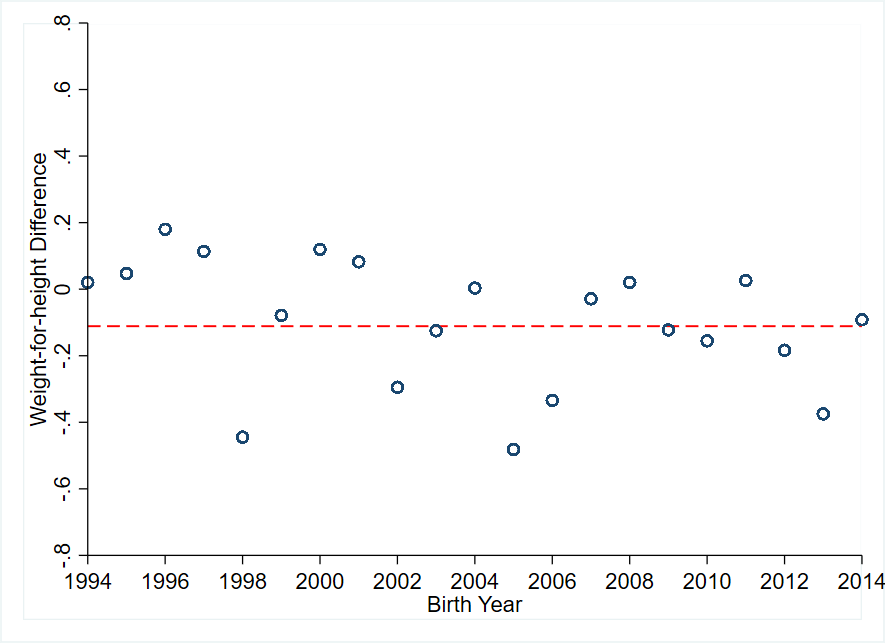
**

Note: For each birth year, the hollow circle represents the average difference in weight-for-height z-scores between children prenatally exposed to droughts and those unexposed. The red dashed line represents the mean value of the weight-for-height difference.

**Figure A4: Weight-for-age Differences by Birth Year**

**
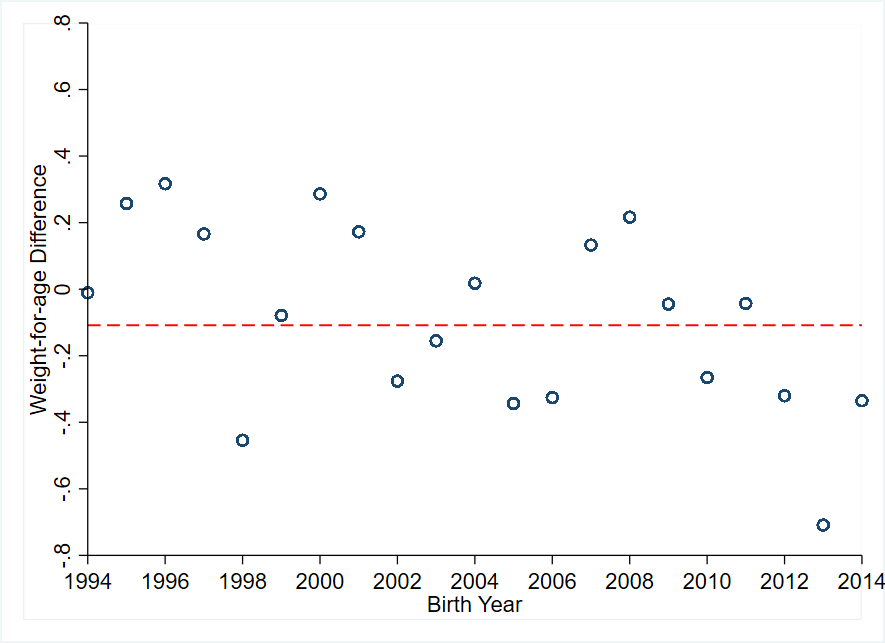
**

Note: For each birth year, the hollow circle represents the average difference in height-for-age z-scores between children prenatally exposed to droughts and those unexposed. The red dashed line represents the mean value of the weight-for-age difference.

| Table A1: Prenatal Exposure to Droughts and Child Health – Additional Heterogeneity Analysis | | | |
| --- | --- | --- | --- |
|  | Height-for-Age | Weight-for-Height | Weight-for-Age |
|  | Z-Score | Z-Score | Z-Score |
|  | (1) | (2) | (3) |
| **Panel A: Male Children** | | | |
| Drought | -0.092* | -0.118*** | -0.115*** |
|  | (0.050) | (0.044) | (0.040) |
| Observations | 14507 | 14507 | 14507 |
|  |  |  |  |
| **Panel B: Female Children** | | | |
| Drought | -0.109* | -0.102** | -0.107** |
|  | (0.063) | (0.041) | (0.043) |
| Observations | 14020 | 14020 | 14020 |
|  |  |  |  |
| **Panel C: First Birth** |  |  |  |
| Drought | -0.101* | -0.147*** | -0.146*** |
|  | (0.063) | (0.051) | (0.055) |
| Observations | 8506 | 8506 | 8506 |
|  |  |  |  |
| **Panel D: Later Birth** |  |  |  |
| Drought | -0.095** | -0.096*** | -0.098*** |
|  | (0.042) | (0.034) | (0.034) |
| Observations | 19906 | 19906 | 19906 |
|  |  |  |  |
| Mother Characteristics | X | X | X |
| Child Characteristics | X | X | X |
| All Fixed Effects | X | X | X |
| Note: *p<0.1, **p<0.05, ***p<0.01. Each column represents the coefficients in a separate regression. The column headings indicate dependent variables. Mother Characteristics consist of mother’s age at birth, mother’s age at birth squared, mother’s years of education, household wealth index, household head gender, whether the household resides in a rural area. Child Characteristics consist of child’s age in months, child’s age in months squared, child’s gender, child’s birth order, as well as whether the child is a singleton birth. All Fixed Effects consist of residential cluster, birth month, and birth year fixed effects. Robust standard errors are clustered at the residential cluster level | | | |

| Table A2: Prenatal Exposure to Droughts and Child Health - Other Outcomes 3 | | |
| --- | --- | --- |
|  | Height (cm) | Weight (kg) |
|  | (1) | (2) |
| Drought | -0.430*** | -0.182*** |
|  | (0.111) | (0.034) |
| Observations | 28,571 | 28,571 |
|  |  |  |
| Mother Characteristics | X | X |
| Child Characteristics | X | X |
| All Fixed Effects | X | X |
| Note: *p<0.1, **p<0.05, ***p<0.01. Each column represents the coefficients in a separate regression. The column headings indicate dependent variables. Mother Characteristics consist of mother’s age at birth, mother’s age at birth squared, mother’s years of education, household wealth index, household head gender, whether the household resides in a rural area. Child Characteristics consist of child’s age in months, child’s age in months squared, child’s gender, child’s birth order, as well as whether the child is a singleton birth. All Fixed Effects consist of residential cluster, birth month, and birth year fixed effects. Robust standard errors are clustered at the residential cluster level | | |
